# Feasibility Study of Thorium-Plutonium Mixed Oxide Assembly In Light Water Reactors

**DOI:** 10.1038/s41598-019-52560-4

**Published:** 2019-11-08

**Authors:** S. S. Mustafa, E. A. Amin

**Affiliations:** 1 Faculty of Science, Zagazig University, Zagazig, Egypt; 2 Nuclear & Radiological Regulatory Authority, Cairo, Egypt

**Keywords:** Physics, Theoretical nuclear physics

## Abstract

Thorium-plutonium mixed oxide, (Th,Pu)OX, is currently used as an alternative fuel in the light water reactors in the world. The main objective of this paper is not only to show the benefits of using the thorium, but mainly to study how the way thorium is introduced in the fuel affects the neutron parameters. Among these benefits is the possibility of extending the operating cycle length and the reduction of the increasing stockpiles of plutonium. The first investigated method is introducing thorium as (Th,Pu)OX. The second one is a homogeneous model of thorium plutonium oxide. It is carried out by adding an amount of plutonium separated from the uranium oxide cycle at 50 GWd/ton of heavy metal to the same amount of thorium. Thus, we studied three assemblies; the reference assembly is uranium oxide of 4.2% enrichment containing borated water as a moderator of concentration 500 ppm (part per million) of B-10. The second is a (Th,Pu)OX and the third one is an assembly with homogenized thorium plutonium. All three assemblies are modeled using MCNPX. A comparison is held between the results of the three lattices. The factors compared are the effective multiplication factor, the inventory of plutonium and uranium isotopes, and the depletion of B-10, the pin by pin power distribution at 0 and 60 GWd/ton and the relative pin radial power for the three lattices. The comparison is aimed to show the effect on the cycle length, the reduction in the Pu content and the power flattening across the assembly. It is found that the evolution of the multiplication factors shows a similar behaviour using (Th-Pu)OX fuel in the assembly as UOX cycle inspite of lowering the K-eff of fresh (Th-Pu)OX fuel (1.19847). The power flattening which is favorable in reactor operation is clearer in (Th,Pu)OX fuel. It is noticed that the mass of Pu-239 decreases by 1.07% from its initial value at the end of life. For homogeneous (Th,Pu)OX, the mass decreases by 0.0832%. The high power peaking factor for (Th,Pu)OX is not expected to cause significant effects during reactor operation but it can be reduced by adding burnable poisons.

## Introduction

The utilization of the Thorium – based nuclear fuel would widely provide fissile resources by breeding U-233. Worldwide resources of thorium are three times larger than uranium resources^1^. Thorium fuel has also several advantages such as reducing the fuel cycle cost, minimizing the U-235 enrichment requirements, and the safety of reactor operation because of lower core excess reactivity requirements. Thorium fuel can be irradiated to higher burnup stages in conditions similar to light water reactors.Th-232 is a fertile material which converts to U-233 due to neutron absorption while other fissile isotopes such as U-235 or Pu isotopes deplete during the fuel irradiation. Th-232 changes rapidly to U-233 that is considered the main fissile material on which the reactor operation depends on^2–4^. In the last decades, the worldwide utilization of light water reactors has produced large amounts of plutonium. This plutonium could be utilized as a new nuclear fuel for future fast reactor systems. The burnup of uranium mixed oxide fuel in the reactor core has been used to reduce the plutonium stockpile, but the uranium in uranium mixed oxide fuels continuously creates new plutonium, making plutonium incineration less efficient. Therefore, other LWR-based methods have been introduced to decrease the plutonium inventory, like mixing plutonium with thorium, which is the topic of this paper^5^.

This study is carried out to investigate the main neutron characteristics of (Th,Pu)OX to show the advantages over traditional low-enriched uranium fuel used in Light Water Reactor. The main advantages are (1) Unlike depleted UOX fuel, (Th,Pu)OX does not produce plutonium (achieving nonproliferation issues in weapons-grade Pu-239 production). (2), (Th,Pu)OX can be irradiated to high burnup values in spite of the low initial value of k-eff. (3) (Th,Pu)OX indicated that the fuel is not only viable, but may also improve the economy of a nuclear power plant by allowing for longer operating cycles and hence a higher availability of the reactor. The good material properties of the thorium-plutonium Mixed Oxide indicate that it has the ability to sustain higher burnups than Uranium-Oxide based fuel types^6,7^. Furthermore, the slow change of the multiplication factor obtained by the depletion of (Th,Pu)OXmakes it possible to achieve high burnups without having an excessively high initial multiplication factor as we will display in the Keff comparisons for the three lattices^8^.

The addition of thorium to currently operating LWRs would result in many different phenomenological impacts on nuclear fuel. Thorium and its irradiation products have different nuclear characteristics than those of uranium. Also, mixing ThO_2_ with UO_2_ or PuO_2_ fuel leads to different chemical and physical properties of nuclear fuel^9^.This paper summarizes some important reactor physics calculations which can predict the reactor safety using comparisons of reference UOX,(Th,Pu)OX and homogeneous (Th,Pu)OX assemblies. The three assemblies have the same parameters, dimensions, and operating conditions except for the loaded fuel.

The main idea of this research begins with the first use of the UOX fuel in the reactor. Then, the separation process of plutonium from the burnt fuel is carried out. Separated plutonium is mixed with thorium and used as a new nuclear fuel in the reactor. This process aims at homogenizing the plutonium in the fuel. This model of homogenized distribution of (Th,Pu)OX fuel was designed by replacing selected rods of the classical UO_2_ assembly by (Th,Pu)OX rods. These selected rods are distributed symmetrically in the assembly. The guide and instrumentation tubes in the assembly remain the same as in the classical UO_2_ assembly.

For carrying out the calculations, MCNPX 2.7 is used for calculating the effective multiplication factors, the pin by pin power distribution, the radial power behavior, the pin peaking factor at BOL and EOL and other important parameters that will be shown in details in the section of results and discussions. Therefore, burn up process consideration of UOX, (Th,Pu)OX and homogeneous (Th,Pu)OX has been proposed in this work using Monte Carol-based transport computational method.

## Calculation Method

MCNPX (MCNP eXtended) is a Fortran90 (F90) Monte Carlo radiation transport computer code that transports nearly all particles at nearly all energies. The version MCNPX 2.7.0 has been used for all the assembly calculations together with the ENDF71X library^10^.The code is used for modeling and simulating the three lattices that are under investigation. The neutron burnup parameters for the three matrices are estimated at the beginning and the end of life. Reflective boundary conditions were applied during the calculations. The calculations were performed for a 17 × 17 PWR fuel assembly with octant symmetry. The assembly included 25 water hole positions. The assembly cans were not considered. The calculations were carried out at a constant power density of 37.7 kW/kg of initial heavy metal and with zero buckling^11^. The depletion process involved the burnup of fuel, uranium oxide or thorium plutonium oxide and Boron-10 of concentration 500 ppm in the light water. All previous works for these models used deterministic codes for the depletion calculations. But, in this work, MCNPX is used for estimating all the burnup parameters. MCNPX results for (Th,Pu)OX are compared with some published results performed by CASMO, MCODE codes and by the participants of the IAEA CRP in ref.^11^. The countries participating in Coordinated Research Programs in this benchmark were Russia, Japan, Republic of Korea, India, and Israel^12^.

### Description of the modeled assemblies by MCNPX Code

**Figure 1:**
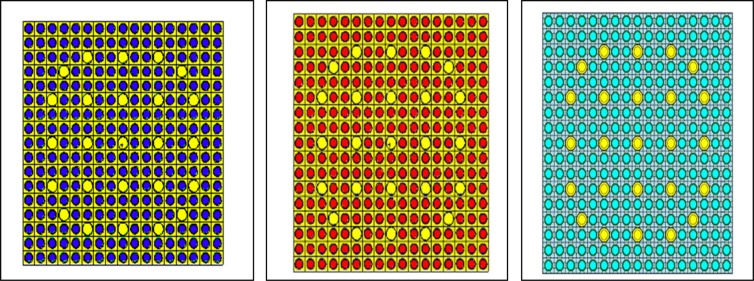
Three models PWR assemblies; (Th,Pu)OX assembly, UOXassembly, homogeneous (Th,Pu) OX performed by MCNPX.

Figure (1) shows three different assemblies, (ThO_2_ 0.095, PuO_2_ 0.05) assembly, UOX assembly of 4.2% U-235 and homogeneous (Th,Pu)OX assembly respectively. The blue rods represent (Th,Pu)OX fuel, the red rods represent UOX fuel and the turquoise rods refer to the homogeneous (Th,Pu)OX fuel which contains plutonium isotopes (Pu-238, Pu-239, Pu-240, and Pu-242) separated from burnt UOX fuel at 50 GWd/ton. The instrumentation and guide tubes containing borated water are represented by yellow color.

The Material compositions of fuel, cladding and moderator are listed in Table (1) for (Th,Pu)OX fuel. The temperature of fuel is 900 K. The cladding material is zirconium; the temperature of the cladding is 600 K. Finally, the moderator is light water with 500 ppm concentration of natural boron and temperature 573 K. The dimensions of the assembly are also listed in Table (2).

**Table 1:** Material compositions (isotope/barn. cm).

Fuel		Cladding	
Th–232	2.0592 × 10^−2^	Zr-natural	4.3241 × 10^−2^
Pu–238	2.2900 × 10^−5^	Moderator	
Pu–239	7.4780 × 10^−4^	H–1	4.7708 × 10^−2^
Pu–240	2.9030 × 10^−4^	O–16	2.3854 × 10^−2^
Pu–241	1.5340 × 10^−4^	B–10	3.9518 × 10^−6^
Pu–242	5.0100 × 10^−5^	B–11	1.5906 × 10^−5^
O–16	4.3710 × 10^−2^

**Table 2:** Geometry of PWR assembly.

Outer dimensions (cm).	22.662 × 22.662
Cell pitch (cm)	1.33306
Fuel pellet radius (cm)	0.4127
Cladding thickness (cm)	0.0617
Equivalent cell radius (cm)	0.7521

The density of uranium oxide is taken as 10.42 g/cm^3^. The burnup calculations from 0 to 60 GWd/ton were executed at a constant specific power of 37.7 W/g HM (of initial heavy metal) for the three lattices. 75 fission products and 24 actinides were tracked in the MCNPX depletion calculations.

## Results and Discussion

### A-Evaluation of multiplication factor versus burnup for thorium plutonium oxide assembly

**Figure 2:**
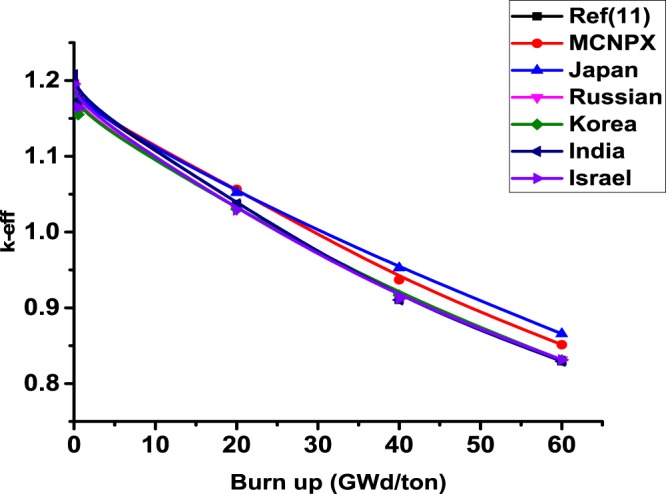
K-effective as a function of burn up in GWd/ton for (Th,Pu)OX assembly predicted by MCNPX code and other codes.

Firstly, the neutronic and burnup analysis of (Th,Pu)OX is introduced. The most important calculated parameter is the neutron multiplication factor on the assembly level. The MCNPX results were compared with other published results in ref.^11^. Figure (2) shows the neutron multiplication factor of the lattice assembly for (Th,Pu)OX fuel as a function of burnup. As it is expected the K-infinity decreases due to the depletion of fissile isotopes and the production of fission products and poisons. Table (3) illustrates the MCNPX results and the results of other participants of the IAEA CRP. The criticality predictions from the various codes agree within about 2.5% at the beginning of life and within 3.5% at 60 GWd/t. The MCNPX results are closer to the results presented by Japan. The beginning and the end of life eigenvalues predicted by MCNPX are slightly lower (by 2.3% and 1.446%respectively) than those predicted by Japan.

**Table 3:** Effective medium neutron multiplication factor versus burnup for MCNPX code.

Burn up GWd/ton	Ref.^11^	MCNPX	Japan	Russian	Korea	India	Israel	CASMO	MCODE
0	1.1852	1.19847 ± 0.00062	1.1987	1.189	1.1864	1.2076	1.1956	1.2035	1.1836 ± 0.0013
0.5	1.1735	1.16719 ± 0.00059	1.167	1.1569	1.1551	1.1736	1.1643	1.1721	—
20	1.0372	1.05649 ± 0.00056	1.0521	1.0298	1.0303	1.0372	1.029	1.0360	1.0233 ± 0.0013
40	0.9104	0.93693 ± 0.00053	0.9527	0.9147	0.9167	0.9104	0.9119	0.9115	0.9124 ± 0.0011
60	0.8294	0.85124 ± 0.00048	0.8657	0.8315	0.831	0.8294	0.8314	0.8188	0.8318 ± 0.0011

The differences between criticality calculations in the previous results are due to the number of fission products and actinides tracked in the used codes for all participants and the cross-section data libraries used in each code. One hundred fission products and 29 actinides were tracked in MCODE calculation while one hundred and eight individual nuclides are considered in CASMO4 depletion calculations.

MCODE utilized primarily ENDF-VI cross-section data. CASMO4 cross-section data are based on evaluated data files JEF-2.2 and ENDF/B-VI that are processed by NJOY-91.91 to generate libraries in 70 energy groups in CASMO4 format.

### B-The variation of Thorium, Plutonium and Uranium isotopes versus burnup for (Th,Pu) OX assembly

**Figure 3:**
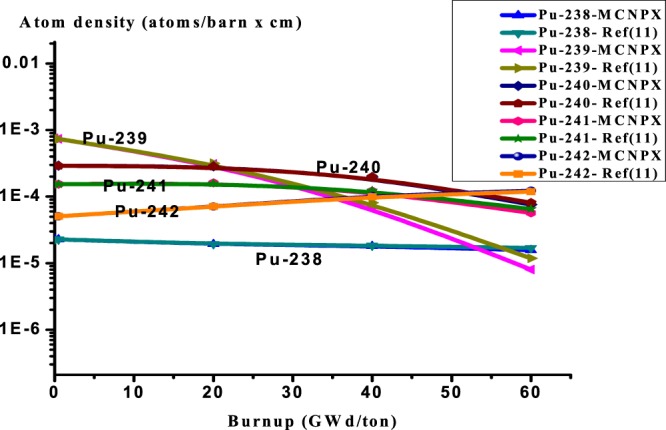
Variation of all plutonium isotopes versus burnup for thorium plutonium oxide assembly. The y-axis is the atom densities on log_(10)_ scale.

The MCNPX results obtained for the composition inventory of the actinides are listed in Table (4) as a function of the heavy metal burnup. It is noted that there is a good agreement of Pu-238 concentration with ref.^11^, IAEA benchmark results, and other participants. In Fig. 3, a reasonable agreement for the even plutonium isotopes, concentrations (Pu-238, Pu-240, and Pu-242) is reached. The odd plutonium isotopes (Pu-239, Pu-241) atom densities are in the range of ref.^11^ results. Finally, an acceptable agreement is found for the isotopes Th-232 and U-233.

**Table 4:** Assembly-average fuel composition (actinides) as a function of burnup.

Burnup GWd/ton	Ref.^11^ results	MCNPX Results	Russian	Japan	Korea	India	Israel
Th-232		Number Density (atoms/barn × cm)					
0	2.06E-02	2.06E-02	2.06E-02	2.06E-02	2.06E-02	2.06E-02	2.06E-02
0.5		2.06E-02	2.06E-02	2.06E-02	2.06E-02		
20	2.04E-02	2.04E-02	2.04E-02	2.04E-02	2.04E-02	2.04E-02	2.04E-02
40		2.01E-02	2.01E-02	2.01E-02	2.01E-02		2.01E-02
60	1.97E-02	1.98E-02	1.98E-02	1.98E-02	1.98E-02	1.97E-02	1.98E-02
Pu-238		Number Density (atoms/barn × cm)					
0	2.29E-05	2.29E-05	2.29E-05	2.29E-05	2.29E-05	2.29E-05	2.29E-05
0.5		2.28E-05	2.28E-05	2.28E-05	2.28E-05		
20	1.83E-05	1.93E-05	1.94E-05	1.95E-05	1.93E-05	1.83E-05	1.94E-05
40		1.80E-05	1.83E-05	1.88E-05	1.80E-05		1.79E-05
60	7.49E-06	1.57E-05	1.69E-05	1.82E-05	1.64E-05	7.49E-05	1.61E-05
Pu-239		Number Density (atoms/barn × cm)					
0	7.48E-04	7.48E-04	7.48E-04	7.48E-04	7.48E-04	7.48E-04	7.48E-04
0.5		7.35E-04	7.35E-04	7.35E-04	7.35E-04		
20	2.99E-04	3.09E-04	3.17E-04	3.27E-04	3.18E-04	2.99E-04	3.15E-04
40		6.97E-05	8.10E-05	9.61E-05	8.20E-05		7.73E-05
60	4.79E-05	8.02E-06	1.18E-05	1.70E-05	1.21E-05	4.79E-05	1.05E-05
Pu-240		Number Density (atoms/barn × cm)					
0	2.90E-04	2.90E-04	2.90E-04	2.90E-04	2.90E-04	2.90E-04	2.90E-04
0.5		2.91E-04	2.91E-04	2.91E-04	2.91E-04		
20	2.85E-04	2.85E-04	2.83E-04	2.68E-04	2.82E-04	2.85E-04	2.85E-04
40		1.97E-04	1.98E-04	1.85E-04	1.99E-04		2.01E-04
60	6.70E-05	7.59E-05	8.09E-05	8.39E-05	8.74E-05	6.70E-05	8.46E-05
Pu-241		Number Density (atoms/barn × cm)					
0	1.53E-04	1.53E-04	1.53E-04	1.53E-04	1.53E-04	1.53E-04	1.53E-04
0.5		1.54E-04	1.54E-04	1.54E-04	1.54E-04		
20	1.55E-04	1.61E-04	1.59E-04	1.70E-04	1.61E-04	1.55E-04	1.58E-04
40		1.21E-04	1.23E-04	1.36E-04	1.23E-04		1.21E-04
60	5.39E-05	5.71E-05	6.50E-05	7.41E-05	6.41E-05	5.39E-05	6.39E-05
Pu-242		Number Density (atoms/barn × cm)					
0	5.01E-05	5.01E-05	5.01E-05	5.01E-05	5.01E-05	5.01E-05	5.01E-05
0.5		5.05E-05	5.05E-05	5.04E-05	5.05E-05		
20	7.20E-05	7.08E-05	7.09E-05	6.81E-05	7.25E-05	7.20E-05	7.02E-05
40		1.02E-04	9.88E-05	9.25E-05	1.04E-04		9.83E-05
60	1.16E-06	1.22E-04	1.19E-04	1.10E-04	1.29E-04	1.16E-04	1.19E-04
U-233		Number Density (atoms/barn × cm)					
0							
0.5		6.86E-07	7.32E-07	7.92E-07	7.38E-07		
20	1.60E-04	1.43E-04	1.52E-04	1.60E-04	0.000154	1.60E-04	1.53E-04
40		2.46E-04	2.61E-04	2.75E-04	2.64E-04		2.68E-04
60	3.19E-04	2.93E-04	3.14E-04	3.31E-04	3.16E-04	3.19E-04	3.24E-04
U-234		Number Density (atoms/barn × cm)					
0							
0.5		2.09E-08	2.36E-08	2.52E-08	1.56E-08		
20	9.63E-05	7.74E-06	8.57E-05	9.71E-06	8.03E-06	9.63E-06	7.91E-06
40		2.43E-05	2.67E-04	2.89E-05	2.52E-05		2.53E-05
60	6.20E-05	4.19E-05	5.32E-04	5.43E-05	2.52E-05	6.20E-05	5.05E-05

**Figure 4:**
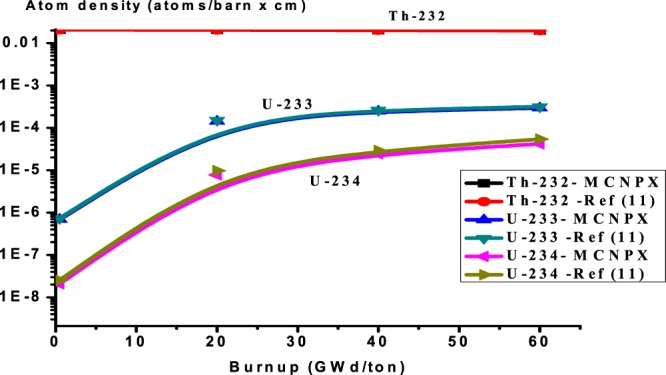
Variation of Th-232, U-233, U-234 versus burnup for thorium plutonium oxide.

Pu-239 decreases with burnup, where its amount nearly has been burnt up at the end of burnup 60 GWd/ton. Pu-241 increases then decreases, this is due to that Pu-241 is produced via the capture of Pu- 240 and on the other hand, Pu-241 is depleted due to fissioning. In Fig. 4, the fissile nuclide U-233 increases with burnup due to neutron capture in Th-232 and subsequent decay of Th-232 to Pa-233 then to U-233 which is fissioned by thermal neutrons.U-234 also increases because it is produced via capture in U-233 and capture in Pa-233 and subsequent decays of Pa-234.

In connection with the uncertainty of some results, the burnup calculations confirm that the MCNPX computer code is suitable for scoping studies of thorium plutonium based fuel designs. It predicts reasonably well the criticality and the composition of the fuel, and their results fall within the uncertainties of the other codes used by the participants in the IAEA international benchmark. This is indicated in Table 3. The values of the uncertainty of MCNPX are lower than those of MCODE. This means that sufficient neutron histories were accumulated to achieve these accurate values

### C-The burnup analysis for the three assemblies

In this section, a comparison is held between the three lattices from the view of the effective multiplication factor, the total average flux, the average energy per fission, the power distribution and pin power peaking factor at the beginning and end of the cycle and the relative radial power at 0, 30 and 60 GWd/ton. All the previous parameters are affected by the variation of Pu-239 and U-233 and this will appear in the discussion of the results.

### C1: The effective multiplication factor

**Figure 5:**
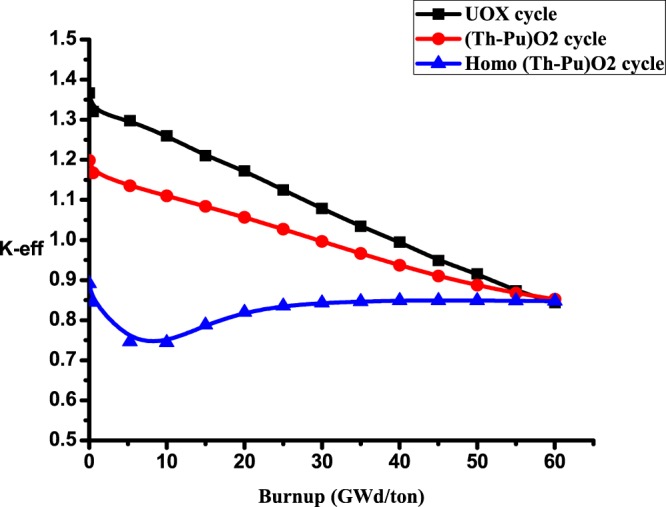
K-effective versus burnup for the three lattices.

As indicated in the Fig. (5), it shows that k-effective for UOX and (Th,Pu)OX fuels decrease with burnup due to the depletion of the fissile isotopes and the release of xenon-135 which has a high neutron capture cross-section. But k-effective for homogenized (Th,Pu)OX is found to be lower than that of UOX at the beginning. Then; it increases due to the buildup of U-233. Finally, it decreases slowly at the end of life due to the depletion of fissile isotopes (formation of low fissile contents). It is noted that the criticality of (Th-Pu)OX is close to that of UOX fuel despite the higher value of K-eff for fresh UOX fuel (1.36699) Therefore (Th,Pu)OX fuel achieves more safety for the reactor operation.

### C2: The average total neutron flux

**Figure 6:**
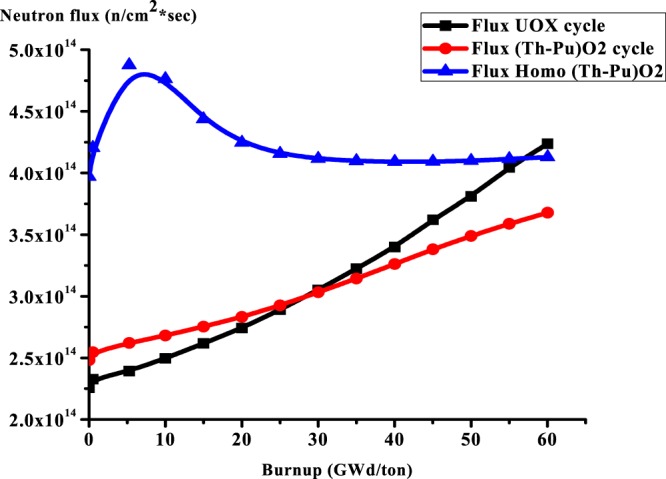
The average total neutron flux versus burnup for the three lattices.

As is shown in Fig. 6, the total average neutron flux for homogeneous (Th,Pu)OX fuel increases with burnup at the beginning because of decreasing the macroscopic fission cross- sections where there is a depletion of fissile nuclides. After that, it decreases with burnup due to the formation of fissile isotopes as U-233, Pu-239, and U-235 that absorb thermal neutrons to be fissioned.

But the total flux increases linearly with burnup for the UOX cycle to compensate for the consumption of the fissile isotopes where the burnup is carried out at constant power. The total average neutron flux for (Th,Pu)OX shows the same behavior of UOX flux but flux flattening is better in (Th,Pu)OX.

### C3: The average energy per fission

**Figure 7:**
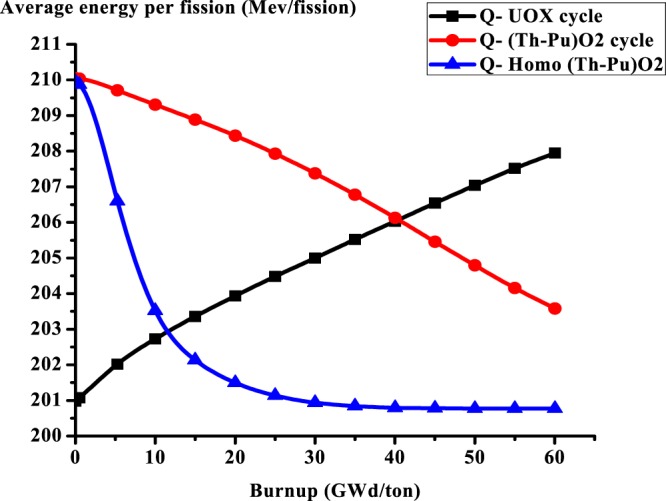
The average energy per fission versus burnup for the three lattices.

As illustrated in Fig. 7, for (Th,Pu)OX homogeneous distribution, the average energy per fission decreases with burnup. This is due to the change of fissile nuclides where the smooth transition from plutonium fissioning to U-233 fissioning causes a decrease in the average energy per fission. This is the result of the fact that the fissile plutonium isotopes release about 200 MeV thermal energy per fission and 2.89 neutrons. But, U-233 only releases about 190 MeV thermal energy per fission and 2.48 neutrons. For the UOX cycle, the energy per fission increases with burnup due to the presence of U-235 which is fissioned by one neutron and releases about 200 MeV per fission and releases around 2.43 neutrons which in turn can initiate other fissions and consequently increasing the average energy per fission.

### C4: The pin by pin power distribution at (0 and 60 GWd/ton)

The Figures from 8–10 show the power distribution shape through the lattice assembly for 1/8 assembly of uranium oxide, 1/8 assembly of homogeneous thorium-plutonium oxide and 1/8 assembly of homogenized thorium-plutonium oxide (in which separated plutonium from burnt uranium oxide at 50 GWd/ton is added to the same amount of thorium) at 0 GWd/ton respectively. The three assemblies use the same parameters, dimensions, and temperatures of fuel, clad and moderator except for the material composition. Published results are compared with MCNPX results for (Th,Pu)OX assembly in Fig. (10).

**Figure 8:**
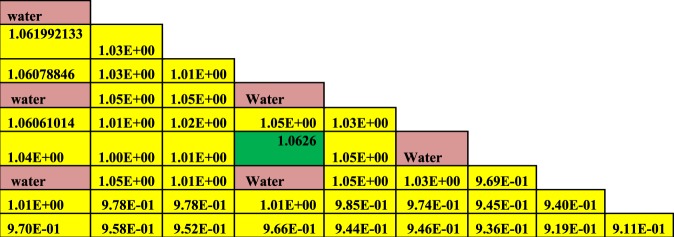
Pin by pin power distribution for UOX assembly with 500 of B-10 at 0 GWd/ton.

**Figure 9:**
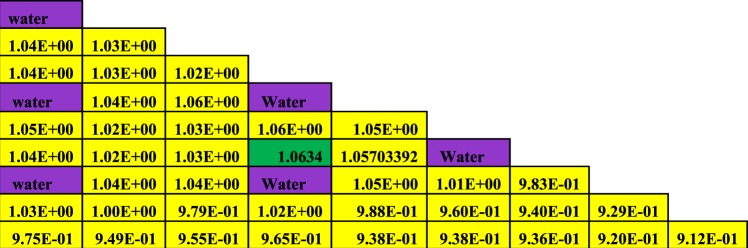
Pin by pin power distribution for homogeneous (Th,Pu)OX assembly with 500 of B-10 at 0 GWd/ton.

**Figure 10:**
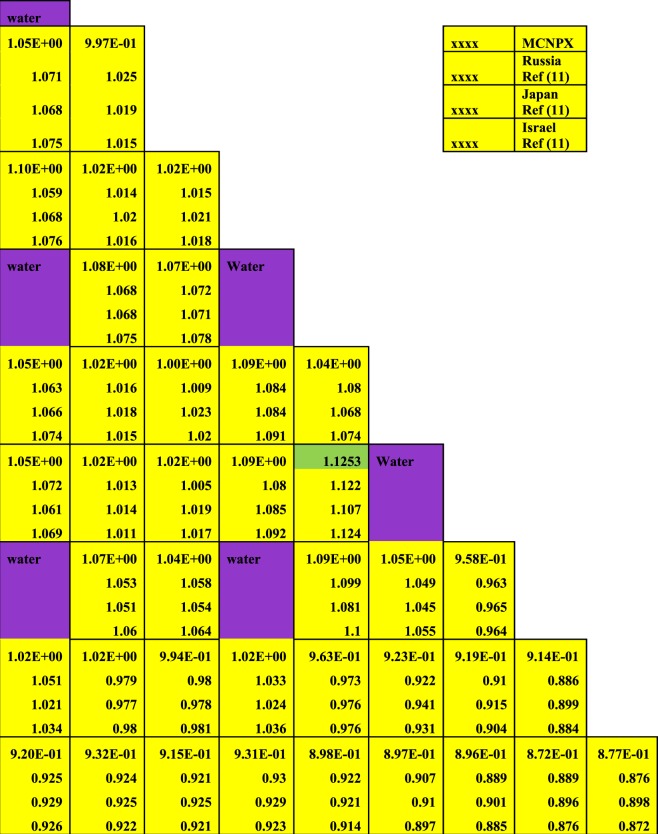
Pin by pin power distribution for (Th,Pu)OX assembly with 500 of B-10 at 0 GWd/ton.

Before analyzing the results, the pin peaking factor is defined as the local pin power divided by the average pin power in the assembly, yielding a measure of the deviation of the pin power from the average in the assembly.

The power peaking factor acts as the bridge between the neutron and thermal-hydraulics analysis of the nuclear reactor core. Because it describes maximum power released locally in the assembly and consequently maximum energy was generated in the fuel rod (at the hot spot) of this assembly. It is used to prevent fuel from reaching a melting point during operation.

Pin power distributions are carried out at T(fuel) = 900 K, T(clad) = 600 K,T(moderator) = 573 K, and C(boron) = 500 ppm.

Figure 8 shows the relative pin power distribution through the lattice assembly of UOX (4.2% enrichment) at 0 GWd/ton under the same circumstances of (Th,Pu)OX assembly. We can see that the maximum relative pin power for UOX assembly is 1.062603147 and the minimum relative pin power is 0.911. The average relative pin power is 0.984.

Figure 9 illustrates the relative pin power distribution for the homogenized (Th,Pu)OX at 0 GWd/TON under the same circumstances of both UOX and (Th,Pu)OX assemblies except the concentration of plutonium added to thorium. The maximum relative pin power for thorium plutonium lattice is 1.063471107 but the minimum relative pin power is 0.912 and the average relative power is 0.985.

Figure 10 depicts the relative pin power distribution for the (Th,Pu)OX at 0 GWd/TON. The maximum relative pin power for thorium plutonium lattice is 1.1253079 but the minimum relative pin power is 0.872 and the average relative power is 1.02.

From these results, it is noticed that the maximum relative and average pin power are higher for (Th,Pu)OX assembly than for homogenized (Th,Pu)OX. The maximum relative and average pin power results for UOX assembly are lower than those for thorium fuels.

As can be observed in Figs 9 and 10, the lattices containing plutonium result have higher power peaking near the guide tubes due to the increased thermal fission cross-section of Pu-239 compared to that of U-235. Generally, the fuels containing thorium result have a slightly higher maximum pin-peaking factors than UOX (i.e., the pin peaking factors for (Th,Pu)OX are higher than those for UOX).

The small increase in pin peaking factors for thorium-bearing fuels is not expected to cause significant issues during reactor operation. However, it is likely that these slightly higher peaking factors could be reduced through enrichment zoning or the implementation of burnable poisons. It can be concluded the pin power peaking increases when (Th,Pu)OX or homogeneous (Th,Pu)OX fuel assemblies are introduced.

**Figure 11:**
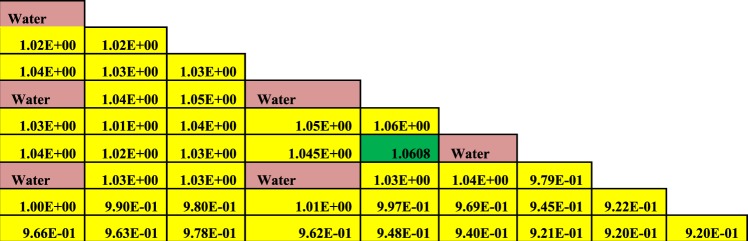
Pin by pin power distribution for UOX assembly with 500 of B-10 at 60 GWd/ton.

**Figure 12:**
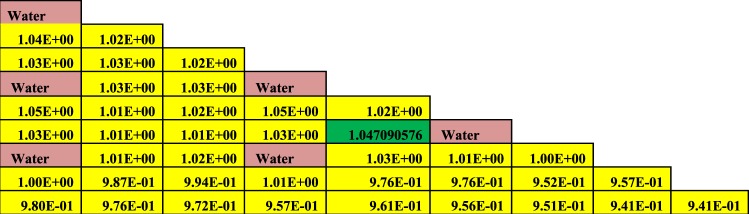
Pin by pin power distribution for homogeneous (Th,Pu)OX assembly with 500 of B-10 at 60 GWd/ton.

**Figure 13:**
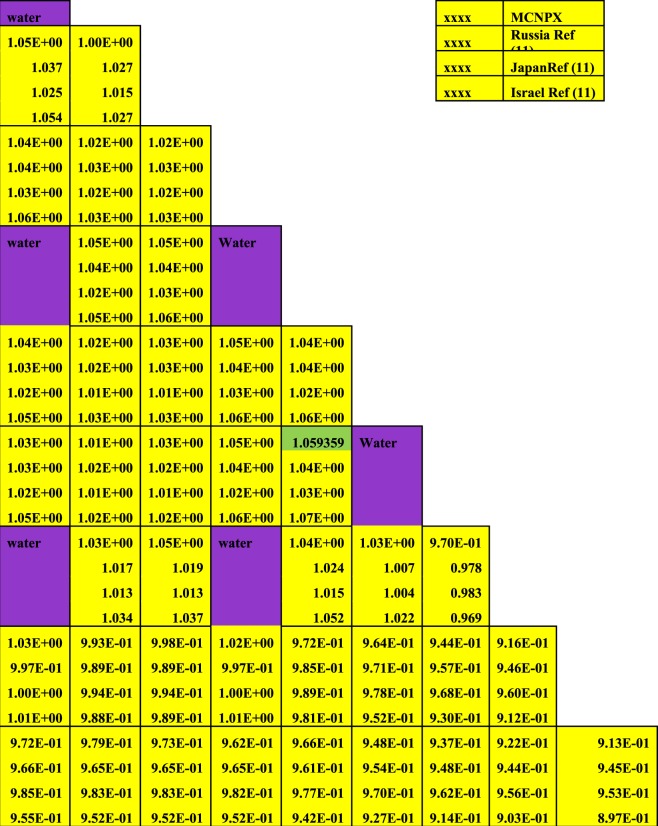
Pin by pin power distribution for (Th,Pu)OX assembly with 500 of B-10 at 60 GWd/ton.

The Figs 11–13 depict the pin by pin power distribution for three lattices at 60 GWd/ton.

From the results of power distribution at the end of life (60 GWd/ton), the maximum relative pin powers are 1.045093, 1.04709 and 1.059359 for UOX, homogeneous (Th,Pu)OX and (Th,Pu)OX respectively. The average relative pin powers are 0.984, 0.989 and 1.02 but, the minimum relative powers are 0.9199, 0.941 and 0.913. Then, both homogeneous (Th,Pu)OX and (Th,Pu)OX fuels have higher maximum relative pin powers than UOX fuel at the end of life of reactor operation

### C4: The depletion of B-10 for the three lattices

**Figure 14:**
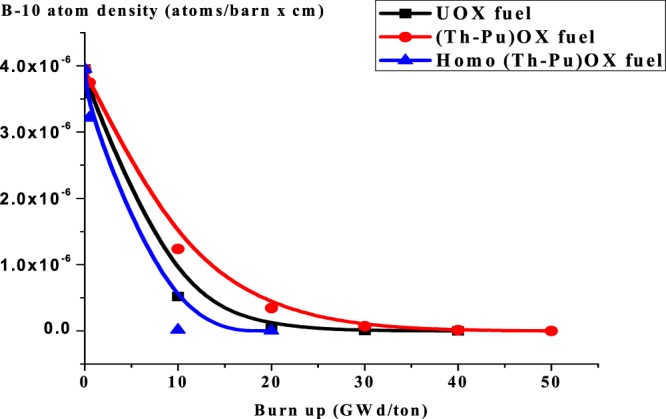
Variation of B-10 atom density in three lattices understudy.

From Fig. (14), B-10 atom density decreases with burnup due to the high thermal absorption cross-section of B-10 that makes it interact with thermal neutrons to produce Helium and lithium which are stable and non –radioactive materials. The presence of Pu-239 – which has higher thermal absorption cross section compared with U-235- in homogeneous (Th,Pu)OX and (Th,Pu)OX contributes to decreasing the thermal neutron absorption efficiency in other materials in the assembly, this means that the inclusion of thorium contributes to a higher thermal absorption cross section, a correspondingly lower thermal neutron flux and boron worth.

### C5: The variation of Pu-239 masses for the three assemblies

**Figure 15:**
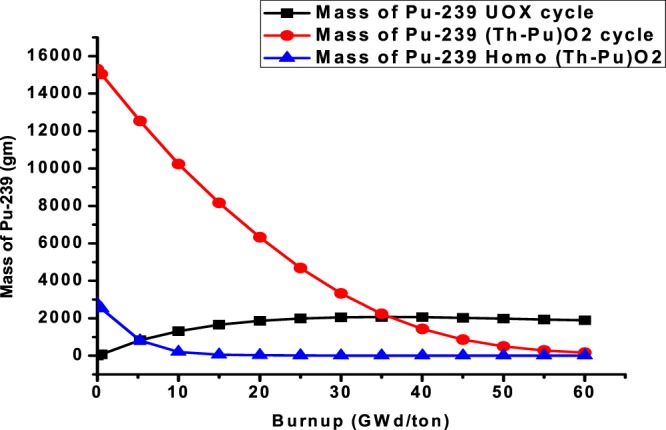
The change of Pu-239 masses for the three assemblies C6: The relative pin radial power distribution at (0,30 and 60 GWd/ton).

One of the most useful benefits of reactors operation with thorium fuel is the decrease of plutonium content in the assembly with burnup. This is indicated in the Fig. (15). The obtained data from the MCNPX code shows that the mass of fissile Pu-239 increases from 0 to 1894 grams for UOX cycle. But it decreases for (Th,Pu)OX cycle from 15310 to 164 grams. For homogeneous (Th,Pu)OX, the mass changes from 2767 to 2.3 grams. The applicable trend is strong for (Th,Pu)OX because Pu-239 decreases to 1.07% of its initial mass at the end of life. For homogeneous (Th,Pu)OX, the mass decreases to 0.0832% of the initial value.

The F4, Fm14 and SD4 tallies in MCNPX are used for evaluating power over each fuel rod. The cross-section of the assembly is assumed symmetrical with respect to the origin. Hence, the radial power distribution is also symmetrical with respect to the origin. The radial power distributions are calculated for selected positions, which are in the left middle row in the assembly.

Figures from 16–18 display the radial power profile in the three assemblies at burnup at 0, 30 and 60 GWd/ton, respectively. The shapes of the profiles are not so regular. The power profiles are figured by calculating the power in twelve fuel cells lying in the middle of the three assemblies. Several spikes appear because of the guide tubes. There is no power output there. It is assumed that power values in the empty guide tubes equate to the nearby values in fuel rod cells. The minimum power values in radial direction occur at the boundary of the assembly due to neutron leakage.

**Figure 16:**
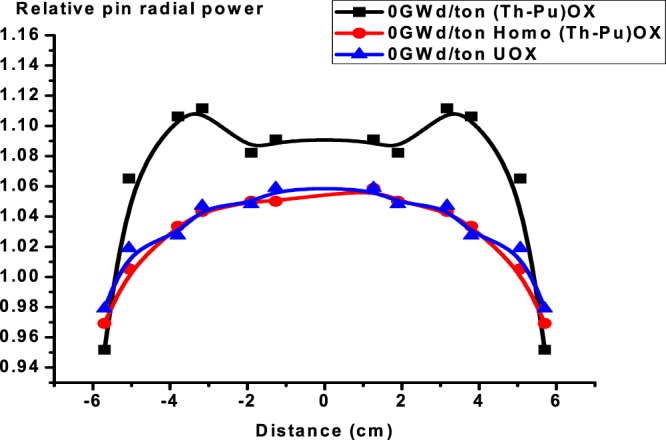
Relative pin radial power distribution at 0 GWd/ton for three assemblies.

**Figure 17:**
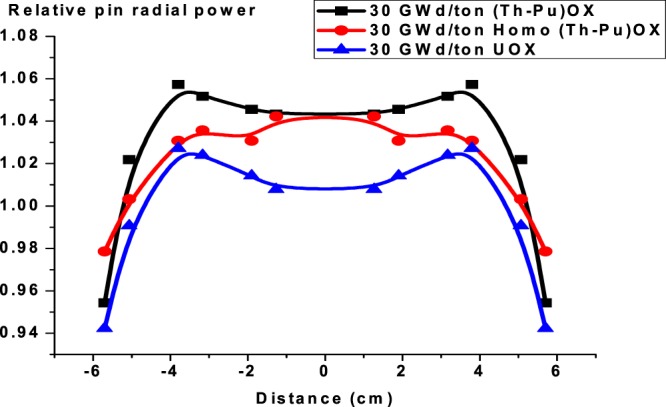
Relative pin radial power distribution at 30 GWd/ton for three assemblies.

**Figure 18:**
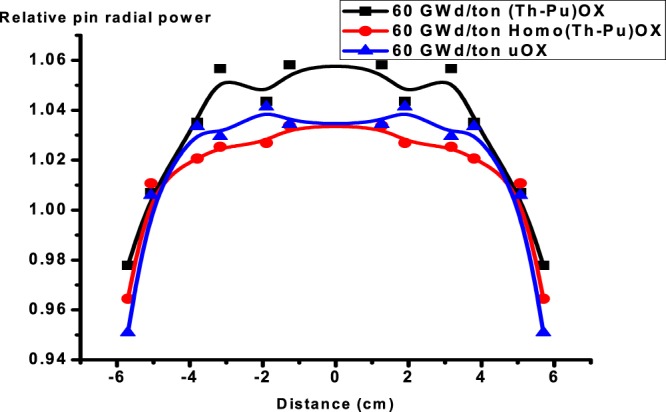
Relative pin radial power distribution at 60 GWd/ton for three assemblies.

The results show that firstly,(Th,Pu)OX assembly has the highest radial power profile in the three stages. This is due to the high absorption cross section of Pu-239 compared to U-235 as stated before. Secondly, the radial power profile is more flat for (Th,Pu)OX or homogeneous (Th,Pu)OX models, but the peaking factor of the peak power pin is almost always greater as we stated before. To mitigate these impacts, it is preferred that some type of burnable absorbers would be needed to reduce power peaking.

The radial power profile for UOX fuel exceeds that of homo (Th,Pu)OX at 60 GWd/ton because of the buildup of Pu-239 and the complete depletion of B-10. These two factors have a significant effect on increasing the power in the fuel rods.

## Conclusion

Many investigations have been focused on the utilization of thorium fuel in nuclear reactors. The present work introduces the investigation of thorium plutonium and uranium oxide fuels usage in a typical light water reactor assembly. The study included the investigation of a thorium plutonium fuel lattice to be used instead of the UO2 fuel alternatives in the light water reactor to achieve non-proliferation issues in weapons-grade Pu-239 production. Analysis has been performed for evaluating the significance and magnitude of reactor safety parameters of interest for three assemblies modeled and simulated by MCNPX code. The calculated and compared values are criticality, fuel composition, total flux, average energy per fission, B-10 concentration and relative pin radial power as a function of burn up. The simulation concluded that (Th,Pu)OX contributes to more power flattening especially at the end of the cycle. Furthermore, this fuel type can be irradiated to high burnup values in spite of lowering the initial k-eff value. Finally, it serves in decreasing the plutonium inventory as the burnup proceeds.
